# Fatty pancreas on EUS: Risk factors, correlation with CT/MRI, and implications for pancreatic cancer screening

**DOI:** 10.1097/eus.0000000000000109

**Published:** 2025-03-03

**Authors:** Ramez M. Ibrahim, Shantanu Solanki, Wei Qiao, Hyunsoo Hwang, Ben S. Singh, Irina M. Cazacu, Adrian Saftoiu, Matthew H. G. Katz, Michael P. Kim, Florencia McAllister, Manoop S. Bhutani

**Affiliations:** 1 Department of Gastroenterology, Hepatology and Nutrition, The University of Texas MD Anderson Cancer Center, Houston, TX, USA; 2 Department of Biostatistics, The University of Texas MD Anderson Cancer Center, Houston, TX, USA; 3 Department of Oncology, Fundeni Clinical Institute, 022328 Bucharest, Romania; 4 Department of Gastroenterology and Hepatology, Elias Emergency University Hospital, Carol Davila University of Medicine and Pharmacy, Bucharest 050474, Romania; 5 Department of Surgical Oncology, The University of Texas MD Anderson Cancer Center, Houston, TX, USA; 6 Department of Clinical Cancer Prevention, The University of Texas MD Anderson Cancer Center, Houston, TX, USA.

**Keywords:** Fatty pancreas, Pancreatic cancer, EUS, computed tomography, magnetic resonance imaging, EUS, Pancreatic steatosis

## Abstract

**Background and Objectives:**

Fatty pancreas (FP), traditionally perceived as a benign finding, has been undergoing scrutiny lately due to growing evidence linking it to various disease states, including increased risk for pancreatic cancer (PC).

**Methods:**

A retrospective study of patients who underwent EUS at a single institution from August 2007 to October 2023, conducted by one endosonographer with more than 25 years of experience. Focusing on individuals identified with FP during EUS, we compared these findings with corresponding findings on computed tomography/magnetic resonance imaging (CT/MRI) conducted within 3 months or 1 year prior to or following EUS.

**Results:**

Ninety-one patients were included and identified as having FP on their EUS exams. The most common indication for EUS was PC screening in high-risk patients (35.16%). At the time of conducting EUS, 65.93% of patients had a body mass index (BMI) ≥30, 63.73% had hypertension, and 32.96% had type 2 diabetes mellitus (DM). Of the 91 patients, 70 had CT or MRI done within 3 months of the EUS date, and only 15 (21.43%) had FP reported on imaging. All 91 patients had CT or MRI within 1 year, and only 16 (17.58%) had FP reported on imaging.

**Conclusion:**

Only 21.43% of patients had FP on their CT/MRI within 3 months despite EUS findings, suggesting either lower accuracy of CT/MRI compared to EUS in identifying FP or potential underreporting in a real-world setting, even in a tertiary care center. This discrepancy in reporting is noteworthy considering FP's role as a potential precursor to several important conditions and promoting pancreatic carcinogenesis pathways.

## INTRODUCTION

Fatty pancreas (FP), also known as pancreatic steatosis or fatty infiltration of the pancreas, has gained increased recognition in recent years for its potential clinical significance after being traditionally perceived as a benign finding.

The history of research on FP traces back to 1933, when Ogilvie provided the first description of pancreatic fat. Ogilvie's comparison of pancreata derived from obese and control cadavers revealed a significantly higher mean pancreatic adiposity in obese cadavers (17.1% *vs.* 9.3%).^[1]^ Subsequent studies, such as Olsen's extensive autopsy study in 1978, reinforced the correlation between pancreatic fat content with body weight and age.^[2]^

In contemporary research, different imaging modalities have been utilized to assess the prevalence of FP. Sepe et al. employed EUS and found a prevalence of 27.8% in their study population^[3]^, whereas Silva et al. utilized trans-abdominal US (TUS) and identified a 12.9% prevalence in healthy subjects without known hypertension or diabetes.^[4]^ FP was also found to be related to age. Pancreatic fat content, along with pancreatic parenchyma volume, increases from birth to age 20 and reaches its highest levels in the third and fourth decades. Beyond the age of 60, pancreatic parenchyma volume declines, resulting in a higher fat-to-parenchyma ratio despite stable total pancreatic fat volume.^[5,6]^

The pathophysiology of FP involves 2 primary mechanisms: fatty replacement and fatty infiltration. Fatty replacement entails the death of pancreatic acinar cells, replaced by adipocytes, triggered by various conditions, including genetic factors such as fibrosis, excessive alcohol consumption, viral infections, iron overload, use of certain medications (such as corticosteroids), or obstruction of the pancreatic duct as seen in chronic obstructive pancreatitis. On the other hand, fatty infiltration involves fatty accumulation within the pancreas, often associated with metabolic syndrome and/or obesity, defining the condition known as nonalcoholic fatty pancreatic disease (NAFPD).

To ascertain whether FP is associated with specific metabolic risk factors and metabolic syndrome, Wu et al. found that 12.9% of 557 healthy individuals without known hypertension or diabetes were diagnosed with FP. They exhibited older age and higher body mass index (BMI), abdominal girth, blood glucose levels, triglycerides, and systolic blood pressure compared to those without FP.^[7]^ Additionally, Sepe et al. found that each 1-unit increase in BMI (OR, 1.05; *P* = 0.03) or fatty liver presence (OR, 3.61; *P* < 0.01) was independently associated with FP on EUS.^[3]^

FP was traditionally perceived as a benign finding. However, as awareness of pancreatic steatosis and its clinical implications increases, recognizing and reporting this entity is important. FP on EUS is an important finding that all endosonographers may not be reporting or looking for. The aim of this study was to review FP diagnosis during EUS and to determine its association with risk factors and demographics, as well as to compare it with its description by other imaging modalities such as computed tomography (CT) or magnetic resonance imaging (MRI) done in the same patient within a short duration. We believe these findings have important implications for pancreatic cancer screening in high-risk individuals (HRIs).

## METHODS

This single-center retrospective study was approved by The University of Texas MD Anderson Cancer Center's institutional review board. A retrospective review was performed for all patients who underwent EUS at The University of Texas MD Anderson Cancer Center between August 1, 2007, and October 30, 2023. All procedures were performed by a single endosonographer (M.S.B.) with over 25 years of expertise in conducting EUS. All the EUS procedures were performed using a linear-array echoendoscope (Olympus GF-UCT180, Tokyo, Japan) at 7.5 MHz.

Patients identified with FP during EUS were included in the analysis. We compared these findings with the corresponding reported findings from cross-sectional imaging conducted within 3 months or 1 year prior to or following EUS, either at the same institution or at other institutions. Patients were excluded if they did not have CT or MRI imaging done within 1 year prior to or following EUS. Clinical and laboratory data, as well as outcomes of interest, were obtained from the electronic medical records.

We categorized patients into 2 categories: patients who had CT/MRI within 3 months prior to or after EUS, and patients who had CT/MRI within 1 year prior to or after EUS. The focus on a 1-year and, more importantly, 3-month time frame for comparison was based on the potential long-term alterations in risk factors for FP. Factors such as weight fluctuations and changes in metabolic syndrome parameters could influence pancreatic fatty content over time.

Patient characteristics were summarized using descriptive statistics, frequency (%) for categorical variables, and median (min, max) or mean for continuous variables. Fisher's exact test for categorical variables and Wilcoxon rank sum test for continuous variables were used to compare the 2 groups.

### Diagnosis of fatty pancreas by EUS in the current study

**Figure 1:**
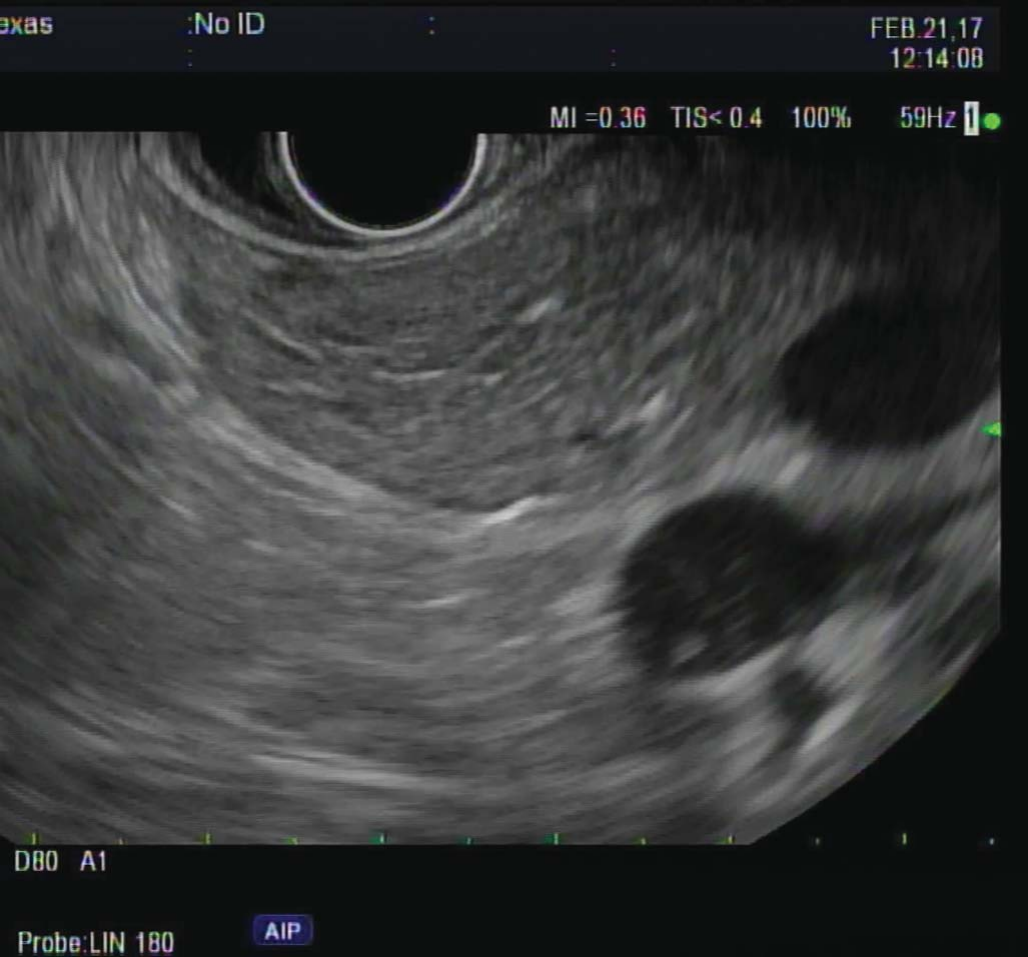
Normal isoechoic “salt and pepper” echo pattern.

**Figure 2:**
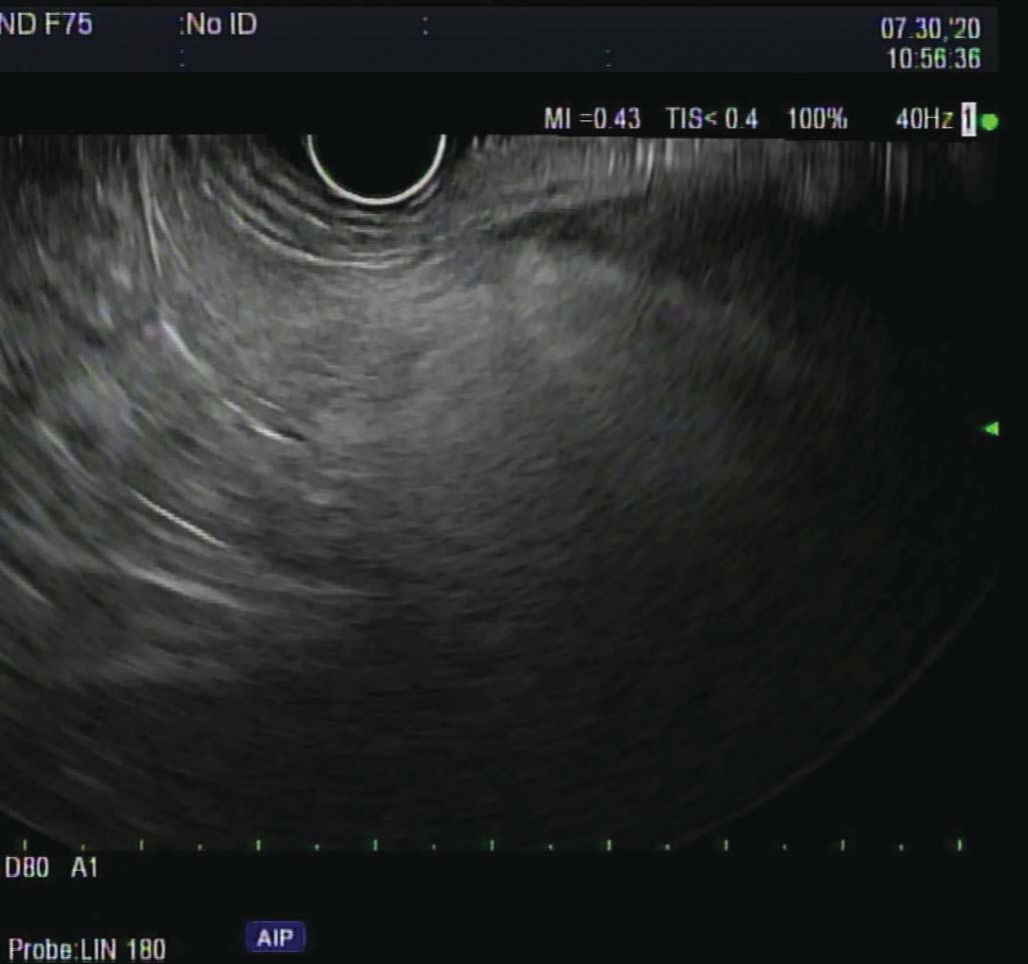
Diffuse fatty pancreas: diffuse hyperechogenicity on EUS.

**Figure 3:**
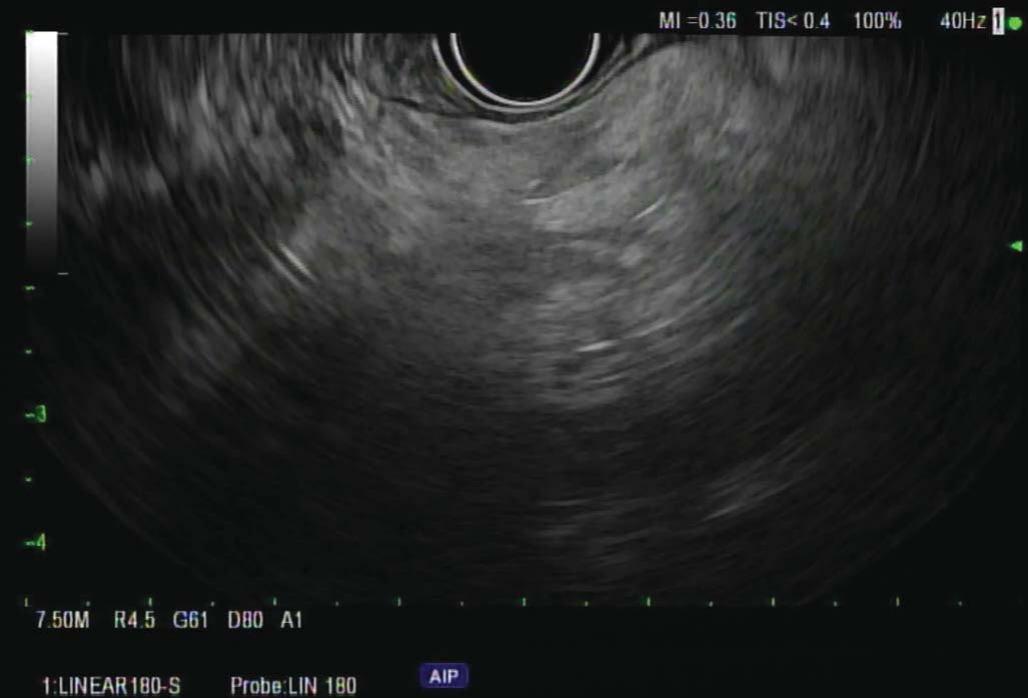
Scattered fatty infiltration in the body of pancreas.

**Figure 4:**
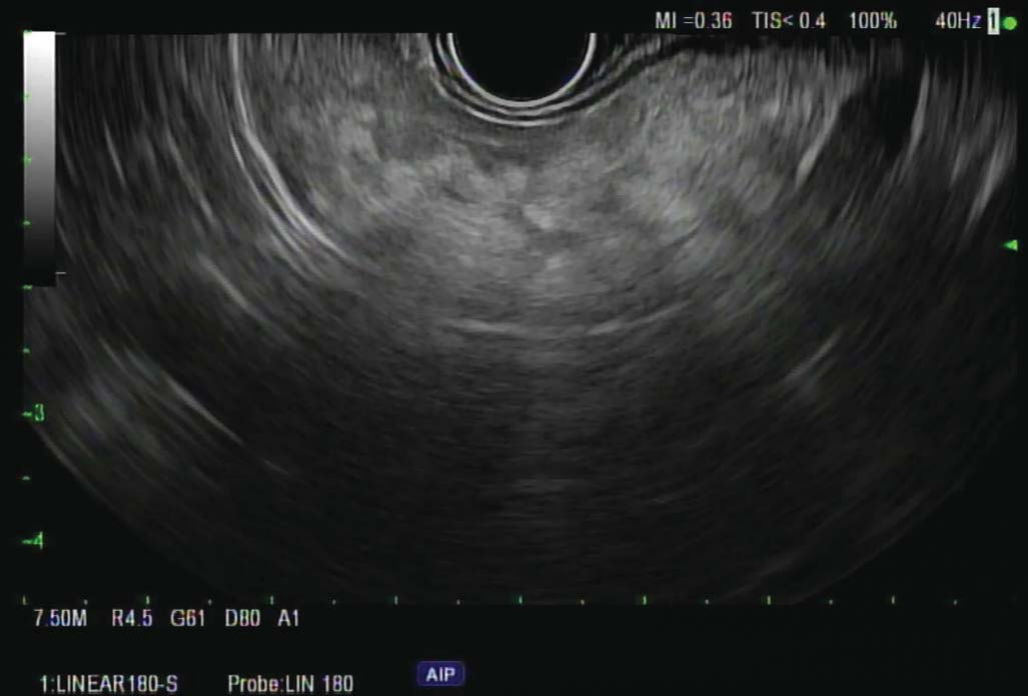
Scattered fatty infiltration in the tail of pancreas.

FP on EUS typically appears as hyperechogenicity of the pancreatic parenchyma compared to its normal isoechoic “salt and pepper” echo pattern [Figure 1]. The hyperechogenicity can be variable in distribution and severity. Some patients have diffuse hyperechogenicity with blurring of the pancreatic margins with no visible main pancreatic duct or blurry margins of the main pancreatic duct [Figure 2]. Others have scattered areas of hyperechogenicity in the pancreas, which may be limited to only the head, body, or tail [Figures 3 and 4]. In patients with scattered hyperechogenicity with patchy fatty infiltration, the pancreas appears heterogeneous. Some parts of the normal pancreas that are spared from fat infiltration can look like a nodule or a tumor, which is a false-positive finding.

## RESULTS

A total of 91 patients were included and identified as having FP during EUS. The indications for performing EUS varied, with the most common being screening for pancreatic neoplasm in high-risk individuals (35.16%), followed by suspected pancreatic masses detected on imaging studies (20.87%). At the time of EUS, it was found that a significant portion of patients had a body mass index (BMI) of 30 or above (65.93%), indicating a prevalence of obesity within the study population. Additionally, a significant proportion of patients were diagnosed with hypertension (63.73%) and type 2 diabetes mellitus (32.96%) [Table 1], further underscoring the prevalence of metabolic comorbidities among individuals with FP. Genetic mutations associated with an increased risk of pancreatic cancer (including BRCA1/BRCA2/ATM/CDKN2A/TP53/PALB2) were present in 27.47% of the individuals included in the study.

**Table 1:** Patient demographics.

Patient characteristics	*N* = 91
Male	56 (61.5%)
White or Caucasian	79 (86.8%)
Hypertension	61 (67.03%)
Dyslipidemia	62 (68.13%)
Social history	
Moderate alcohol use	49 (53.8%)
Severe alcohol use	4 (4.4%)
Former smoker	30 (32.96%)
Current smoker	5 (5.49%)
Patient characteristics at the time of EUS	*N* = 91
Age (mean)	61.81
Hypertension	58 (63.73%)
Type 2 diabetes	30 (32.96%)
BMI (mean)	32.66
Overweight (25–29.9)	25 (27.47%)
Obese class I (30–34.9)	34 (37.36%)
Obese class II (35–39.9)	15 (16.48%)
Obese class III (40+)	11 (12.08%)
Indications for conducting EUS	
Screening for pancreatic mass	32 (35.16%)
Suspected pancreatic mass on imaging	19 (20.87%)
PCN follow-up	15 (16.48%)
Other	25 (27.47%)
Gene mutation	
BRCA2	12 (13.18%)
BRCA1	4 (4.39%)
PALB2	3 (3.29%)
ATM	2 (2.19%)
TP53	2 (2.19%)
CDKN2A	1 (1.09%)
BRCA1/BRCA2	1 (1.09%)

BMI, body mass index; PCN, pancreatic cystic neoplasm.

Further analysis of cross-sectional imaging reports was done. Among the 91 patients, 70 had undergone CT or MRI done within 3 months before or after their EUS procedure, and only 15 (21.43%) were reported to have FP on their imaging during this time frame. Moreover, when extending the imaging time frame to 1 year before or after EUS, all 91 patients underwent CT or MRI, with only 16 patients (17.58%) being reported to have FP on their imaging scans [Table 2].

**Table 2:** Locating fatty pancreas on EUS *vs*. CT/MRI.

Fatty pancreas findings on EUS, *n*	91
CT/MRI done within 3 months of EUS, *n*	70
• Fatty pancreas findings on imaging, *n* (%)	15 (21.43%)
• NO fatty pancreas findings on imaging, *n* (%)	55 (78.57%)
CT/MRI done within 1 year of EUS, *n*	91
• Fatty pancreas findings on imaging, *n* (%)	16 (17.58%)
• NO fatty pancreas findings on imaging, *n* (%)	75 (82.4%)
• Fatty liver findings on imaging, *n* (%)	32 (35.16%)

In our investigations into factors affecting the detection of FP on CT/MRI, we conducted a comparative analysis of demographic data concerning the subset of 70 patients who underwent CT/MRI within 3 months prior to or following EUS, in terms of concordance and discordance. The concordance group included cases where FP was reported on both EUS and CT/MRI, and the discordance group included cases where FP was solely detected on EUS. Table 3 offers a detailed examination of patient characteristics, allowing for a comprehensive comparison. Our analysis revealed no statistically significant differences in patient characteristics, suggesting a potential issue of underreporting or underdiagnosis rather than variations in patient demographics.

**Table 3:** Comparison of patient characteristics, CT/MRI done within 3 months (fatty pancreas: *N* = 70).

		Total (*N* = 70)	Discordance (*N* = 55)	Concordance (*N* = 15)	P*
Age, median (min, max)		63 (32, 83)	63 (32, 79)	63 (40, 83)	0.83
Gender, no. (%)	Female	26 (37.14%)	20 (76.92%)	6 (23.08%)	>0.99
	Male	44 (62.86%)	35 (79.55%)	9 (20.45%)	
Hypertension, no. (%)	No	21 (30%)	18 (85.71%)	3 (14.29%)	0.53
	Yes	49 (70%)	37 (75.51%)	12 (24.49%)	
Dyslipidemia, no. (%)	No	21 (30%)	15 (71.43%)	6 (28.57%)	0.36
	Yes	49 (70%)	40 (81.63%)	9 (18.37%)	
ETOH use, no. (%)	Moderate	35 (50%)	28 (80%)	7 (20%)	0.89
	No or minimal.	32 (45.71%)	24 (75%)	8 (25%)	
	Severe	3 (4.29%)	3 (100%)	0 (0%)	
Diabetes at EUS, no. (%)	No	47 (67.14%)	35 (74.47%)	12 (25.53%)	0.35
	Yes	23 (32.86%)	20 (86.96%)	3 (13.04%)	
BMI, no. (%)	Normal	6 (8.57%)	3 (50%)	3 (50%)	0.08
	Obese class I	27 (38.57%)	19 (70.37%)	8 (29.63%)	
	Obese class II	11 (15.71%)	10 (90.91%)	1 (9.09%)	
	Obese class III	8 (11.43%)	6 (75%)	2 (25%)	
	Overweight	18 (25.71%)	17 (94.44%)	1 (5.56%)	
Indications for conducting EUS, no. (%)	Other	17 (24.29%)	14 (82.35%)	3 (17.65%)	0.11
	Pancreatic cystic neoplasm	11 (15.71%)	9 (81.82%)	2 (18.18%)	
	Pancreatic solid mass	2 (2.86%)	2 (100%)	0 (0%)	
	PNET	4 (5.71%)	2 (50%)	2 (50%)	
	Screening for pancreatic neoplasm	18 (25.71%)	17 (94.44%)	1 (5.56%)	
	Suspected mass	18 (25.71%)	11 (61.11%)	7 (38.89%)	
Gene mutation, no. (%)	BRCA1/BRCA2/ATM/CDKN2A/TP53/PALB2	16 (22.86%)	15 (93.75%)	1 (6.25%)	0.16
	MEN1/MEN2a	3 (4.29%)	3 (100%)	0 (0%)	
	NO	51 (72.86%)	37 (72.55%)	14 (27.45%)	

*P value calculated by the Wilcoxon rank sum test for continuous variables and by the Fisher's exact test for categorical variables. A *P* value <0.05 is considered significant.

BMI, body mass index; PNET, pancreatic neuroendocrine tumors.

## DISCUSSION

In our study focusing on consecutive cases of FP identified through EUS, we observed a notable prevalence of obesity, hypertension, type 2 diabetes, and dyslipidemia among these cases. These findings align with prior research linking FP to these comorbidities.

In a retrospective cross-sectional study by Khoury et al., 78 out of 569 patients who underwent EUS for hepatobiliary indications were identified as having FP. Both univariate and multivariate analyses revealed significant associations between FP and metabolic syndrome parameters (obesity, hyperlipidemia, and liver steatosis).^[8]^ Similarly, in a study by Wang et al. involving 8079 Chinese subjects, a 16% prevalence of FP was found, with higher rates of diabetes (12.6% *vs.* 5.2%), NAFLD (67.2% *vs.* 35.1%), hypertension (12.7% *vs.* 7.1%), low-HDL cholesterol (39.% *vs.* 27.1%), and hypertriglyceridemia (36.2% *vs.* 20.2%) among individuals with FP compared to those without FP (*P* < 0.001).^[9]^

Although comorbidities can be risk factors for FP, as mentioned above, they can be consequences too. Chan et al. conducted a 10-year prospective cohort study to investigate the metabolic outcomes of FP. Compared to the non-FP group, the FP group had a higher incidence of diabetes mellitus (DM), hypertension, and dyslipidemia during long-term follow-up evaluation. Moreover, each percentage increase in pancreatic fat escalated the risk of incident diabetes by 7%.^[10]^

A recent study has found that among middle-aged individuals at high risk of Alzheimer's dementia, increased pancreatic fat levels in males, but not females, was associated with lower global cognition and reduced brain volume. This finding underscores the significance of FP and its potential implications.^[11]^

FP has been linked to both acute and chronic pancreatitis, although the potential cellular mechanisms underlying this association remain not fully understood. Notably, a study demonstrated a higher prevalence of FP among patients with a history of pancreatitis compared to those without (37.7% *vs.* 4.7%). Further analyses, both univariate and multivariate, underscored a significant correlation between FP and a history of acute pancreatitis.^[12]^ Another investigation suggested that an increased level of pancreatic steatosis, as indicated by a lower pancreas-to-spleen attenuation ratio, may correlate with the severity of acute pancreatitis.^[13]^

A recent study showed that the presence of FP on CT was a risk factor for post-ERCP pancreatitis, and it was suggested that prophylactic measures such as rectal nonsteroidal anti-inflammatory drugs (NSAIDs) be used before ERCP in these patients, as this may lower the risk of post-ERCP pancreatitis.^[14,15]^ Similarly, detecting and reporting FP on an EUS conducted prior to an ERCP could be an important factor in considering rectal NSAIDs for preventing post-ERCP pancreatitis. This stresses the need for endosonographers and radiologists to report the presence of FP on EUS or cross-sectional imaging as an important finding.

FP has important implications not only for pancreatitis but also for pancreatic cancer (PC), a correlation that might not be widely appreciated, especially by endosonographers or radiologists. The relationship between FP and PC has been a subject of many studies, although the exact nature of their association is not fully understood. One hypothesis is that FP shares common risk factors with PC, such as obesity. Obesity is known to be associated with both FP and an increased risk of PC. Stolzenberg-Solomon et al. reported a 45% increased risk for PC in individuals with a BMI of ≥35.^[16]^ Similarly, Arslan et al. demonstrated a positive correlation between increased BMI and PC risk.^[17]^ A meta-analysis done by Aune et al. further confirmed this link, revealing a risk ratio of 1.10 for every 5-unit BMI increment.^[18]^

Furthermore, some studies have focused on the direct relationship between FP and PC. Hori et al.^[19]^ observed a significantly higher degree of fatty infiltration (FI) in pancreatic ductal adenocarcinoma (PDAC) cases compared to controls (OR, 6.1; *P* < 0.001), whereas Rebours et al. identified a connection between intralobular fat and pancreatic intraepithelial neoplasia (PanIN) (OR, 17.86; 95% CI, 4.935–88.12).^[20]^ In a retrospective study, Khoury et al. found that FP was significantly associated with PC (OR, 2.62; 95% CI, 1.23–5.57; *P* = 0.01) based on EUS examinations in patients with hepatobiliary indications.^[21]^ These findings collectively highlight the strong association between obesity, FP, and the risk of PC.

Lesmana et al. conducted a study involving 162 patients who underwent EUS, revealing PC in 26.5% and FP in 32.7% of subjects, with a notable overlap in PC patients exhibiting FP. Assessing various factors, including age, gender, diabetes, and chronic pancreatitis, the study identified FP as the sole significant risk factor for PC.^[22]^ This observation prompted a crucial consideration regarding the potential utility of EUS as a screening tool for the early detection of pancreatic malignancy in NAFPD patients. In a recent prospective study involving 42,599 participants, researchers discovered that about 17.86% of them had FP. Over a medium follow-up period of 4.61 years, 782 individuals developed new-onset pancreatic diseases. The study revealed a significant relationship between intrapancreatic fat deposition (IPFD) and the incidence of both exocrine pancreatic diseases such as acute pancreatitis, and PC, as well as endocrine pancreatic diseases, including DM.^[23]^ Additionally, Khoury et al. highlighted a higher prevalence of main-duct intraductal papillary mucinous neoplasm (MD-IPMN) in the FP group (10.3%) compared to those without FP (3.3%), suggesting a potential indirect link to PC given MD-IPMN's high malignancy risk.^[8,24]^

FP was also found to be a notable risk factor for the development of postoperative pancreatic fistula (POPF) after pancreatectomy. The leakage of pancreatic secretions can cause serious adverse events, including peritonitis, sepsis, hemorrhage, malnutrition, chronic pancreatitis, or death. Pancreatic fat infiltration can increase the softness of the pancreatic gland, which can lead to the occurrence of POPF. This association underscores the importance of detecting FP early in patients with PC or at high-risk of developing PC. This allows for proactive measures to reduce the level of fatty infiltration and enables surgeons to take precautions during pancreatic surgery to prevent adverse events.^[25,26]^

To answer the question of whether the increased intrapancreatic fat in the pancreatic ductal adenocarcinoma (PDAC) cases, compared to controls, could be a process that is primary or secondary to tumor-associated inflammation, Desai et al. found that there is no difference in the fatty infiltration of the pancreas (FIP) before and after the diagnosis of PDAC in these cases. This result suggests that pancreatic steatosis, rather than resulting from cancer-associated inflammation, is a carcinogenic risk factor.^[27]^

The gold standard for diagnosing FP is histological examination, which accurately detects pancreatic fat content and reveals histological changes such as increased adipocytes and intracellular triglycerides. However, due to its invasive nature, histology is primarily reserved for research purposes and is not feasible for routine clinical use.^[28]^

Various imaging modalities are employed for detecting FP, each with distinct strengths and limitations. Transcutaneous ultrasound (TUS) compares pancreatic echogenicity with the liver, kidney, and spleen, labeling a comparatively hyperechoic pancreas as an FP. However, TUS is subjective and operator-dependent, affected by factors like imaging settings and patient anatomy. Furthermore, hepatic echogenicity can be altered by steatosis, which can further complicate the interpretation of pancreatic echogenicity.^[5]^ EUS offers superior resolution and can detect subtle changes, but it is invasive, requires specialized expertise, and may not be widely accessible. A hyperechoic pancreas is considered synonymous with FP. CT scans are easily accessible and cost-effective. Pancreatic attenuation typically decreases with fatty infiltration on unenhanced images. However, CT has ionizing radiation and may misinterpret hypoattenuating masses or cysts as focal fatty changes.^[29]^

MRI is considered the most accurate noninvasive modality for diagnosing FP, particularly when utilizing advanced techniques such as magnetic resonance proton density fat fraction (MR-PDFF) and magnetic resonance spectroscopy (MRS). MRI's superior soft tissue contrast, the absence of ionizing radiation, and its ability to precisely quantify fat deposition make it an ideal tool for assessing the severity of fat infiltration. However, despite these advantages, the application of these advanced MRI techniques is limited in routine clinical practice. Their high costs, restricted availability, and the requirement for specialized expertise pose significant barriers to widespread use.

Our findings reveal a significant discrepancy in the reporting of FP on CT/MRI compared to EUS, prompting an important question: does this difference suggest potential limitations in the accuracy of cross-sectional imaging compared to EUS, or might it indicate underreporting?

In terms of accuracy, EUS is a high-resolution imaging modality with the ability to detect small and occult tumors not visible on CT/MRI scans, as well as to define fine chronic pancreatitis features in the ducts and parenchyma. Thus, EUS would be expected to show FP accurately when present. However, due to its invasive nature, it is typically reserved for cases with clear indications such as high-risk pancreatic cancer screening, the presence of mass or cystic lesions on cross-sectional imaging or ultrasound, or indications related to esophageal or gastric pathologies. Additionally, there may be interobserver variability in reporting FP as EUS is an operator-dependent procedure. Nevertheless, many studies have relied on cross-sectional imaging for FP detection, with reported prevalence rates comparable to other studies that relied on EUS.

In recent decades, liver steatosis has received considerable attention and is frequently reported on cross-sectional imaging, given its link to liver cirrhosis, which can lead to liver cancer, making radiologists attentive to its presence. Conversely, FP is less frequently reported on CT/MRI scans. Given FP's emerging association with other serious comorbidities, including PC, recognized as the most lethal cancer in the world with a very low survival rate and the worst prognosis, and its association with other serious comorbidities, heightened attention to FP during cross-sectional imaging interpretation is crucial. This also underscores the importance of endosonographers, particularly those with limited experience, becoming familiar with the appearance of pancreatic steatosis on ultrasound.

An important finding in our study was the presence of FP in 35.16% of patients undergoing EUS for pancreatic mass screening. It is crucial to note that FP has the potential to conceal an underlying tumor, making it challenging to detect pancreatic neoplasms on EUS. Therefore, it is important to emphasize weight loss interventions and improvements in other metabolic syndrome parameters for individuals with FP, particularly those at higher risk of PC due to genetic mutations or family history. Individuals at high risk for PC are particularly motivated to take measures to reduce their risk of PC or improve early detection. Educating these individuals about the implications of FP on EUS or cross-sectional imaging may provide even greater incentive and motivation for them to adopt lifestyle measures to lose weight and improve other metabolic parameters.

The limitations of the study primarily stem from its retrospective nature. Furthermore, we relied solely on cross-sectional imaging reports without undergoing review by radiologists.

In conclusion, our study of patients with FP identified on EUS revealed that only 21.43% of patients had FP reported on their CT/MRI within 3 months. This suggests either a lower accuracy of CT/MRI compared to EUS in identifying FP or potential underreporting in a real-world setting, even in a tertiary care center. This discrepancy in reporting is noteworthy given the role of FP, or pancreatic steatosis, as a potential precursor to severe conditions, including diabetes, pancreatitis, pancreatic exocrine insufficiency, and pancreatic carcinogenesis. FP on EUS was frequently seen during EUS screening for PC in high-risk patients. This has important implications because FP is a potential risk factor for PC and might also potentially conceal an underlying occult neoplasm on EUS. Furthermore, our study also again shows the strong association of FP with features of metabolic syndrome, such as increased BMI, hypertension, hyperlipidemia, and type 2 diabetes.
